# Child abuse during the COVID-19 pandemic in Bangladesh: a brutal reality

**DOI:** 10.11604/pamj.2021.40.267.31722

**Published:** 2021-12-30

**Authors:** Mosharop Hossian, Md Utba Rashid, Mohammad Hayatun Nabi, Mohammad Delwer Hossain Hawlader

**Affiliations:** 1 Public Health Professional Development Society (PPDS), Dhaka, Bangladesh,; 2 Nutrition and Clinical Services Division (NCSD), International Centre for Diarrhoeal Disease Research, Bangladesh (icddr,b), Mohakhali, Dhaka 1212, Bangladesh,; 3 Department of Public Health, North South University, Dhaka 1229, Bangladesh

**Keywords:** Child abuse, COVID-19, Bangladesh

## To the editors of the Pan African Medical Journal

Violence against children, ranging from mental torture to forceful rape, is a critical public health issue that stifles growth. Convention on the Rights of the Children defined violence against children as “all forms of physical or mental violence, injury and abuse, neglect or negligent treatment, maltreatment or exploitation, including sexual abuse” [1]. Along with all pre-existing forms mentioned in the above definition, online harassment added a new dimension to child abuse during the pandemic situation. Every year, globally, almost one out of every two children or one billion children experience some form of violence [2]. But it is difficult to determine the situation of Bangladesh as there is a dearth of exact statistics related to violence against children. However, as stated by a recent Multiple Indicator Cluster Surveys (MICS) report, nearly 89% of Bangladeshi children aged 1-14 years had experienced violence against them, including physical torture, psychological hostility, etc., from their caregivers in the last one month before the MICS survey conducted [3]. According to KidsRights Index 2021, Bangladesh ranked 110^th^ among 182 countries regarding how children´s rights are respected, although the position was 108th in the pre-pandemic situation. Presumably, the problem is worsening, as there has been a sharp increase in child rape and online harassment during the COVID-19 period [4].

Based on reports from Ain o Salish Kendra (ASK), a Bangladeshi legal aid and human rights organization, at least 6,514 children (including 705 victims aged six years or below) experienced some form of violence within the years of 2016 to 2020 in Bangladesh, but only 3,237 victims sought for legal action [5]. Among all types of reported violence against children, rape incidents were the highest. Analyzing the child rape incidents from 2013 to 2019, we observed a downward trend between 2017 and 2018, which became abruptly in peak in 2019 (562). This trend is escalating even during this pandemic situation. To be specific, 654 child rape cases were reported during 2020, and for the first six months of 2021, the number was 222 [6]. Furthermore, the statistics mentioned above were based on girls only. It will surely increase a lot if we include the boys too. During the COVID-19 pandemic, online child harassment appears to be a major concern. ASK commenced a survey in 2020 among 108 participants randomly selected from the five districts of Bangladesh. The survey results suggested that at least 30% of the children experienced different forms of online harassment, including sexual exploitation, leaking private information, blackmailing, etc., which was nearly 3.5 times higher than the study conducted by ASK among the girls during the pre-pandemic state. ASK also reported that 88% of the online harassment incidents occurred by strangers. But the most alarming fact was that only 6% of the victims had taken legal action [7]. The complex judicial system and lack of awareness among the parents and children might have led to such legal help-seeking behavior.

However, the number of reported cases by different humanitarian organizations are likely a small part of all the incidents that took place and disproportionately represent some of the most discussed occurrences in the country; for instance, 21 out of 222 rape cases resulted in death during the first six months of 2021 [6]. Nevertheless, they shed some light on the extremity of violence against a child, including child rape and online harassment in Bangladesh.

In response to the increasing trend of child abuse cases, the Bangladesh government in October 2018 approved a bill (Amendment of children act, 2013) to establish a child-oriented judicial system with provision to institute one or more child-friendly tribunals in each district of the country [8]. However, the trend of child rape incidents from 2020 to 2021 indicated that only bill approval for child protection was inadequate to change the scenario [6]. Moreover, in the existing laws, pedophilia and child sexual harassment are not adequately addressed. For example, no specific provision is mentioned regarding pedophilia in the Digital Security Act (2018) [9]. Although child pornography has been inscribed in the Pornography Control Act (2012), no legal provision is explicitly mentioned for the incidence where both sufferers and perpetrators are minor [10]. That is why it becomes necessary for policymakers to take more realistic initiatives against brutal child rape and online harassment, for instance, amendment of the law (where required), developing prevention plans, and campaigns to support the sufferers. In this context, humanitarian organizations can play a pivotal role as the helping hand of the government. Their existing setup and experience will definitely make it possible to implement decided prevention plans and campaigns successfully. We also need to remember that the child is isolated and often confined with potential perpetrators during this ongoing situation. Therefore, the guardians should be more cautious about taking care of their children during the pandemic. Besides, there should be mass media coverage to encourage more reporting towards child violence, which will eventually help us reform the social views by strengthening the system to reduce the overall burden.
